# Synemin Redefined: Multiple Binding Partners Results in Multifunctionality

**DOI:** 10.3389/fcell.2020.00159

**Published:** 2020-03-17

**Authors:** Mary A. Russell

**Affiliations:** Department of Biological Sciences, Kent State University at Trumbull, Warren, OH, United States

**Keywords:** synemin, AKAP, PKA, intermediate filament, signal transduction

## Abstract

Historically synemin has been studied as an intermediate filament protein. However, synemin also binds the type II regulatory (R) subunit α of protein kinase A (PKA) and protein phosphatase type 2A, thus participating in the PKA and phosphoinositide 3-kinase (PI3K)-Akt and signaling pathways. In addition, recent studies using transgenic mice indicate that a significant function of synemin is its role in signaling pathways in various tissues, including the heart. Recent clinical reports have shown that synemin mutations led to multiple cases of dilated cardiomyopathy. Additionally, a single case of the rare condition ulnar-mammary-like syndrome with left ventricular tachycardia due to a mutation in the synemin gene (SYNM) has been reported. Therefore, this review uses these recent studies to provide a new framework for detailed discussions on synemin tissue distribution, binding partners and synemin in disease. Differences between α- and β-synemin are highlighted. The studies presented here indicate that while synemin does function as an intermediate filament protein, it is unique among this large family of proteins as it is also a regulator of signaling pathways and a crosslinker. Also evident is that the dominant function(s) are isoform-, developmental-, and tissue-specific.

## Introduction

Synemin is an unusual and exotic intermediate filament (IF) protein that has functions which extend past the normal role of IF proteins. The protein structure of synemin is unusual due to the head domain being shorter and the tail domains (of α and β) being longer than most members of the IF family of proteins (Titeux et al., 2001; Xue et al., 2004). Synemin is considered “exotic” since it is the only cytoplasmic IF representative currently identified that undergoes alternative splicing (Herrmann and Aebi, 2004; Welch et al., 2010). Another unusual characteristic of synemin is its wide tissue distribution. While most IF proteins are used as differentiation markers due to their cell- or tissue-type specific expression, synemin is widely expressed among a variety of tissues. Additionally, IF proteins are found at discrete locations in the plasma membrane such as in costameres or focal adhesions where they serve as a molecular link (Capetanaki et al., 2007; Jones et al., 2017). Again, in atypical fashion for an IF protein, synemin is found in other (non-junctional) membrane locations in astrocytoma cells and cardiac myocytes (Jing et al., 2005; Russell et al., 2006; Pan et al., 2008; Lund et al., 2012). These differences between synemin and other IF proteins are suggestive that synemin has additional functions, and, indeed, it has additional binding partners that are not IF proteins. In fact, two binding partners are signaling molecules; thus, suggesting of a role in their associated signaling pathways [i.e., the protein kinase A (PKA) and phosphoinositide 3-kinase (PI3K)-Akt signaling pathways] (Russell et al., 2006; Pitre et al., 2012). In addition, studies using transgenic mice underscore the importance of synemin in multiple signaling pathways, including the PKA and PI3K-Akt pathways, particularly in bone and muscle tissue (Li et al., 2014; Garcia-Pelagio et al., 2015, 2018; Moorer et al., 2016). Further evidence of the functional importance of synemin includes reports of mutations in synemin leading to heart disease and a rare condition that affects multiple tissues including bone and heart (ulnar-mammary-like syndrome) (Zeller et al., 2006; Ware et al., 2016; Zhang et al., 2018; Zlotina et al., 2018). Taken together these findings indicate synemin is an important regulator of multiple signaling pathways and its functions are clinically significant.

This review strives to accomplish 3 tasks: (1) to describe synemin in light of its initial characterization as an IF protein; (2) to provide detailed information on synemin’s binding partners; and (3) to offer a fresh outlook on the function of synemin by highlighting new information gleaned from studies using knockout mice and diseases caused by synemin mutations.

## Identification of Synemin as in Intermediate Filament Protein

Synemin was first identified as an IF-associated protein (IFAP). It was isolated from chicken smooth and skeletal muscle in association with desmin and vimentin at the Z-disks (Granger and Lazarides, 1980). However, cloning of the synemin gene (*SYNM*) revealed it to be a bona fide IF protein, as it contains the rod domain that allows IF proteins to form coiled-coils dimers which are assembled into 10 nm filaments (Becker et al., 1995; Bellin et al., 1999; Herrmann and Aebi, 2004). While only one isoform of synemin is expressed in chicken, three isoforms are differentially expressed in mammals; α-/high (H, 180 kDa); β-/medium (M, 150 kDa); and, low (L, 41 kDa) (Titeux et al., 2001; Xue et al., 2004). Even though chicken synemin is most similar to the largest mammalian isoform, it only displays 37% overall sequence identity. Due to this low sequence homology between orthologs, human synemin was first identified as a novel gene and named desmuslin (Mizuno et al., 2001) which was later found to be β-synemin (Titeux et al., 2001).

## Structure of Synemin Protein

**FIGURE 1:**
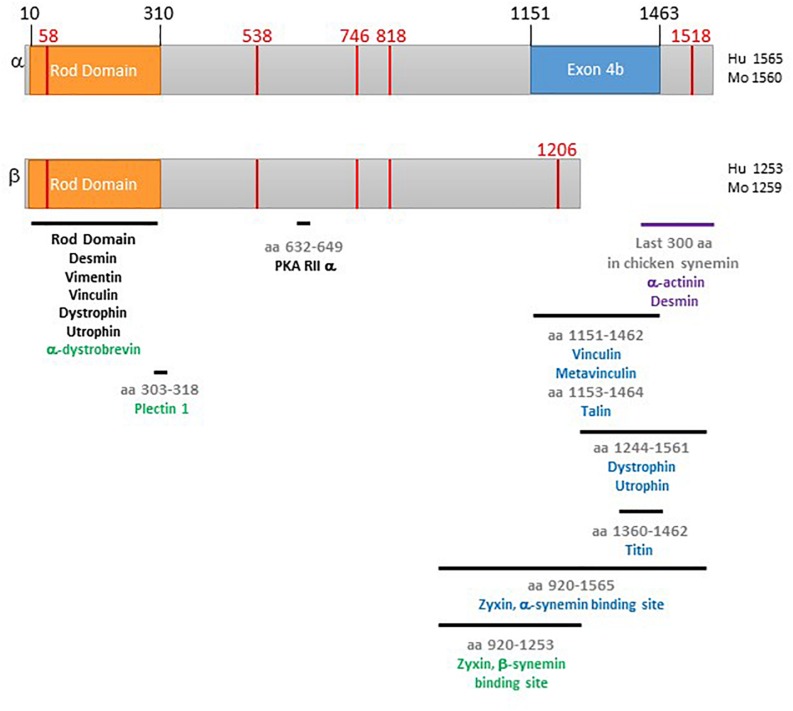
Schematic of α- and β-synemin. The top diagram is α-synemin and the bottom is β-synemin. The rod domain is shown in orange. The exon found only in α-synemin, exon 4b, is in blue. Binding regions for synemin binding partners are indicated by black bars. A **black** protein name indicates that interaction between that protein and **both isoforms** has been demonstrated for that region. A blue protein name indicates that only interaction with α-synemin has been demonstrated for that region. A green protein name indicates that only interaction with β-synemin has been demonstrated for that region. The red vertical lines indicate locations corresponding to mutations causing disease. The purple bar and names indicate the work demonstrating interaction was done with chicken synemin. Numbering of the figure is based on the following GenBank reference protein sequences: NP_663780 (human α-synemin), NP_056101 (human β-synemin), NP_964001 (mouse α-synemin), and NP_997546 (mouse β-synemin).

IF proteins have three domains: a head domain, a central rod domain, and a tail domain. The approximately 310 amino acid central rod domain is able to form the alpha-helical coiled-coil used in forming IFs. IF head and tail domains are of varying lengths (Geisler and Weber, 1982; Quax-Jeuken et al., 1983). Both α- and β-synemin have an unusually short head domain (10 aa) and unusually long C-terminal tails compared to most other IF proteins (Bellin et al., 1999; Titeux et al., 2001). These two differ by the inclusion of one exon in α-synemin [4b, as illustrated in Izmiryan et al. (2010)] which encodes a 312 aa insert near the end of the C-terminal tail (Figure 1). A recently identified isoform, L synemin, shares the head and rod domain of α- and β-synemin. However, fusion of exon 3 to exon 5 results in the use of a different open reading frame, producing a distinctive, smaller, C-terminal tail (Xue et al., 2004). Most research, including ours, is carried out on α- and/or β-synemin, thus, these isoforms will be the primary focus of the rest of this review.

## Synemin as an If Protein

IF networks are one of three structural components of the cytoskeleton along with microtubules and actin microfilaments. The IF super family is expressed in all metazoan cells in a cell- and tissue-specific dependent manner. The function of IFs varies with cell type and ranges from cell motility to signal transduction (Fuchs and Weber, 1994; Coulombe and Wong, 2004; Herrmann and Aebi, 2004; Eriksson et al., 2009; Lowery et al., 2015). IF proteins are grouped into six types (I-VI) based on sequence homology, gene structure and tissue distribution (Herrmann and Aebi, 2000, 2016; Sanghvi-Shah and Weber, 2017). In the past, synemin, together with nestin, tanabin, and transitin have been classified as type VI IFs due to sequence similarity and genomic organization (Steinert et al., 1999; Titeux et al., 2001; Guerette et al., 2007), but more recently this group has been folded into the type IV neurofilament IFs due to being similar in gene structure and also to only being found in vertebrates (Herrmann and Aebi, 2000; Peter and Stick, 2015).

Synemin is not able to form homopolymeric IFs due to deficiencies in the head and rod domain (Khanamiryan et al., 2008), instead it forms heteropolymers usually with type III IF proteins in a tissue-dependent manner. Synemin’s IF binding partners (and non-IF binding partners) are discussed in detail below.

## Synemin Binding Partners

The diversity among C-terminal tails of is thought to provide functional differences among the IF proteins (Guerette et al., 2007; Herrmann and Aebi, 2016). That coupled with the fact that the two large synemin isoforms differ only in the C-terminal tail argues that the functional differences are due to this structural difference. This difference appears to lead to differences in subcellular distribution and/or binding partners.

In support of differential subcellular distribution, α-synemin was found primarily at the sarcolemma and intercalated discs (i.e., sites of cell-cell contact) while β-synemin localized primarily at the Z-disks in neonatal cardiac myocytes (Lund et al., 2012). Synemin’s multiple binding partners within costameres (discussed in the section Binding Partners at Costameres/Focal Adhesions) are likely responsible for localization of synemin to these junctional membrane locations. Synemin localized in non-junctional membrane was shown to co-localize with desmin and α-actinin in neonatal cardiac myocytes and U-373 MG cells (human glioblastoma cells), respectively (Jing et al., 2005; Pan et al., 2008; Lund et al., 2012).

Many studies have focused on investigating synemin binding partners and some have identified isoform specific binding. Below, the plethora of α- and β-synemin binding partners and the subcellular locations of each isoform is discussed. Figure 1, a schematic of α- and β-synemin, illustrates the current state of knowledge as to the region(s) of interaction on each synemin isoform for their binding partners.

### IF Network Binding Partners

IF networks in striated muscle localize to Z-disks maintaining lateral alignment of myofibrils (Paulin et al., 2004; Capetanaki et al., 2007). As previously mentioned, synemin forms obligate heteropolymeric IFs, usually with a type III IF protein. For example, its copolymerizing partner in developing and mature muscle is **vimentin** and **desmin**, respectively (Granger and Lazarides, 1980; Bilak et al., 1998; Bellin et al., 1999; Mizuno et al., 2001). In desmin knock-out mice, synemin is no longer found at the Z-disks in skeletal muscle (Carlsson et al., 2000). Synemin binds desmin and vimentin via the rod domain (Bellin et al., 2001; Mizuno et al., 2001; Figure 1). Both synemin isoforms have the ability to interact with desmin and vimentin as demonstrated with transfection studies and co-immunoprecipitation analysis (Mizuno et al., 2001; Titeux et al., 2001; Jing et al., 2007; Hijikata et al., 2008).

Synemin was shown to co-localize with glial fibrillary acidic protein (GFAP), another type III IF protein, in astrocyte progenitor cells and astrocytoma cells, hepatic stellate cells, and non-myelin-forming Schwann cells (Hirako et al., 2003; Jing et al., 2005; Uyama et al., 2006). *In vitro* blot overlay and co-immunoprecipitaion studies further suggested IF network formation between GFAP and synemin (Hirako et al., 2003; Uyama et al., 2006). However, research with GFAP null mice suggests that synemin does not form IFs with GFAP in astrocytes, instead using vimentin as its polymerizing partner in these cells. Rather, it interacts with GFAP as an IFAP (Jing et al., 2007).

One study has shown that in myoepithelial cells synemin may form heteropolymers with type II IF proteins, the basic cytokeratins. Immunofluorescence microscopy showed that synemin co-localizes to the keratin cytoskeleton; additionally, anti-synemin antibody co-immunoprecipitated both cytokeratin 5 and 6 but not acidic cytokeratin 14 (IF type I) (Hirako et al., 2003).

#### Role of Synemin as an IF Protein

The functional role of synemin as an IF protein has recently been addressed via transgenic animal studies. Because synemin is a minor component of IF filaments (i.e., in a ratio of ∼50:1 with vimentin and ∼25:1 with desmin) (Granger and Lazarides, 1982; Hirako et al., 2003) it is not surprising that synemin null mice displayed minor skeletal and cardiac phenotypes related to IF networks. For example, desmin localized correctly in mature striated muscle (Li et al., 2014; Garcia-Pelagio et al., 2015, 2018). One study did find minor sarcomere abnormalities in soleus muscle in old mice which might be attributed to the loss of synemin from the IF network (Li et al., 2014). However, a different study detected no sarcomere changes in tibialis anterior muscle (Garcia-Pelagio et al., 2015). These differences could be due to the methods used to disrupt the synemin gene, different types of skeletal muscle studied [soleus (slow) vs. tibialis anterior (fast)] or the specific length of fibers chosen to study, each group focused on fibers with different average sarcomere length.

Synemin is not restricted to IF networks, it is found in multiple other locations in various cell types. It has been most extensively studied in muscle, but studies in various cells of the nervous system, in particular astrocytes, and other cell types have also been described. At these other locations synemin is found in association with non-IF binding partners.

### Signaling Molecules as Binding Partners

IF proteins have recently been recognized as multivalent scaffolding proteins. This is exciting because it potentially allows synemin and other IF proteins to act upon various cellular activities including signal transduction (Coulombe and Wong, 2004; Eriksson et al., 2009). Accordingly, it has been shown that synemin regulates two signaling pathways: the PKA and the PI3K-AKT pathways (Russell et al., 2006; Pitre et al., 2012).

#### The PKA Pathway (Synemin Is an AKAP)

A region in the C-terminal tail common to α- and β-synemin is able to bind the regulatory (R) subunit of PKA type IIα (aa 632-649, Figure 1; Russell et al., 2006), this classifies synemin as an AKAP. AKAPs are a large structurally diverse family which share a small motif that allows them to bind to the regulatory subunit of PKA. Upon activation of PKA by binding of cAMP to the R subunits, the C subunits then dissociate and phosphorylate nearby substrates (Scott, 1991). PKA has broad substrate specificity yet is highly selective in a physiological setting. Anchoring of PKA near its substrates via AKAPs is one mechanism used to enhance specificity of the pathway. The ability of AKAPs to tether PKA to specific subcellular locations near physiological substrates is thought to confer specificity on multiple PKA signaling pathways operating in the same cell; in this way different PKA pathways are initiated via a different receptors and end in different physiological outcomes in the same cell (Lester and Scott, 1997; Carnegie et al., 2009). Additionally, many AKAPs are multivalent scaffolds, binding other proteins such as upstream activators like adenylyl cyclase, downstream repressors like phosphatases and phosphodiesterases, and members of other signaling pathways like PKC (Welch et al., 2010; Calejo and Tasken, 2015; Torres-Quesada et al., 2017). This co-localization of multiple signaling pathway participants is thought to allow AKAPs to not only enhance specificity, but to also amplify and accelerate signaling, and to reduce basal activity of PKA (Feliciello et al., 2001; Greenwald and Saucerman, 2011).

In the heart, AKAP-mediated anchoring of PKA regulates contractility (Fink et al., 2001). To date, at least 17 AKAPs and their associated splice variants have been identified in cardiac myocytes (Kritzer et al., 2011). Stimulation of the β-adrenergic pathway in cardiac myocytes results primarily in activation of PKA, including the AKAP-anchored pools distributed to discrete locations throughout the cardiac myocyte near PKA substrates. This allows for the catalytic subunit to phosphorylate various targets including the L-type Ca^+^ channel, ryanodine receptor, phospholamban, troponin I, and myosin binding protein C (Brum et al., 1984; Yoshida et al., 1992; Zhang et al., 1995; Weisberg and Winegrad, 1996; Karczewski et al., 1997). These PKA substrates work in concert upon activation to increase the force of contraction and rate of relaxation. To date, the specific role of synemin-anchored PKA is not known.

#### The PI3K-AKT Pathway

Synemin is expressed in astrocyte progenitor cells but not mature astrocytes (Sultana et al., 2000). It is re-expressed in these cells, however, under pathophysiological conditions, in reactive astrocytes and glioblastoma tumors as discussed in the section Changes in Synemin Expression: Astrocytes (Jing et al., 2005; Pan et al., 2008). RNAi experiments in multiple human glioblastoma cell lines revealed synemin enhances the migratory properties of astrocytoma cells and plays a role in enhancing glioblastoma cell proliferation through its effects on the PI3K-Akt signaling pathway (Pan et al., 2008; Pitre et al., 2012). This pathway is critical for controlling the G1/S transition and thus proliferation (Liang and Slingerland, 2003).

Under normal conditions PI3K can be stimulated by various extracellular signals such as growth factors and hormones (Shi et al., 2019). Upon stimulation, PI3K converts phosphatidylinositol 4,5-bisphophate (PIP2) into phosphatidylinositol 3,4,5-triphosphate (PIP3) (Rubashkin et al., 2014). This results in recruitment of Akt (also called protein kinase B) and phosphoinositide-dependent kinase 1 (PDK1) to the membrane. PKD1 phosphorylation of Akt leads to partial activation. Complete activation is accomplished by phosphorylation at a second site by mTOR complex 2 (Sarbassov et al., 2005; Yu and Cui, 2016). Active Akt prevents accumulation of two cyclin-dependent kinase inhibitors (CDKIs), p21^Cip1^ and p27^Kip1^ (Li et al., 2002; Vervoorts and Luscher, 2008; Lee and Kim, 2009). In the absence of CDKIs, the cell cycle is able to progress from G1 to S due to cyclins binding to and activating cyclin-dependent kinases (CDKs) which phosphorylate retinoblastoma (Rb), a tumor suppressor protein. Once phosphorylated, Rb is no longer able to inhibit transcription of several genes needed to enter S phase (Knudsen and Knudsen, 2008).

Reducing synemin in glioblastoma cell lines led to decreased phosphorylation of Akt and Rb and an increase in protein levels of p21^Cip1^ and p27^Kip1^. This was accompanied by a reduction of cell proliferation due to a decrease in cells moving past the G1/S transition. These results led Pitre et al. (2012) to investigate the PI3K-Akt pathway. They found α- and β-synemin bind to protein phosphatase type 2A (PP2A), the phosphatase primarily responsible for dephosphorylation of the Akt residues hypophosphorylated in these studies (Ruvolo, 2016). They proposed the role of synemin in these cells is to bind PP2A and sequester it away from Akt. Thus, a loss of synemin led to dephosphorylation and inactivation of Akt which allowed accumulation of the CDKIs p21^Cip1^ and p27^Kip1^ leading to inhibition of CDKs and reduction of Rb phosphorylation and loss of progression through the cell cycle. The PP2A binding site on synemin has not been determined.

#### Role of Synemin in Signaling Pathways

Transgenic animal studies emphasize the importance of synemin in signaling pathways. In skeletal muscle, knockout mice displayed increased hypertrophy in response to overload and a decreased rate of fatigue compared to controls (Li et al., 2014; Garcia-Pelagio et al., 2015). The changes in synemin null mice were ascribed to a host of changes in gene expression and post-translational modification. Specifically, analysis of gene expression by PCR revealed decreased expression of two negative regulators of muscle mass (atrogin and myostatin) and increased expression of two positive regulators of muscle mass (follistatin and muscle-specific IGF1) (Li et al., 2014). These were accompanied by an increase in total protein as well as phosphorylated forms of several proteins that play a role in muscle hypertrophy (PKA RIIα, Akt, CREB1, and ribosomal protein S6). The reduced tibialis anterior and quadriceps muscle fiber size seen in mice lacking synemin was also attributed to these changes in signaling proteins (Garcia-Pelagio et al., 2015).

The role of synemin as an AKAP is highlighted by the fact that PKA activity was increased in mice lacking synemin (Li et al., 2014). The authors suggest that the role of synemin may be to hold PKA inactive in skeletal muscle. This role for synemin as an AKAP would explain why a loss of synemin in null mice resulted in increased PKA activity which then resulted in an increase in phosphorylation of PKA substrates (RIIα, CREB1, and ribosomal protein S6). The increase in Akt phosphorylation in skeletal muscle in synemin null mice is in contrast to the reduction seen in glioblastoma cell lines treated with synemin shRNA. The difference might be due to tissue-type specificity of synemin and/or PP2A functions (Ruvolo, 2016) or different experimental models (cell lines vs. transgenic mice).

One study has focused exclusively on the role of synemin in the heart using synemin null mice (Garcia-Pelagio et al., 2018). The resulting phenotype in old mice, a mixture of changes associated with both hypertrophic and dilated cardiomyopathy, illustrates the important yet complex role of synemin in the heart. For example, an increase in the size of cardiac myocytes, in the diastolic pressure, and in left ventricular mass indicated hypertrophic cardiomyopathy. However, a decrease in left ventricular ejection fraction and fractional shortening indicated dilated cardiomyopathy. Therefore, a loss of synemin resulted in heart disease in old mice, even young mice displayed reduced left ventricular contractility. The physiological results of impaired ejection and contractility correlate with cellular results in synemin null mice; a decrease in magnitude of sarcomere shortening and a decrease in Ca^+^ transient amplitude. The authors speculate that the most likely cause of the mixed phenotype is due to changes in either PKA or extracellular signal-regulated kinase 1/2. Western blot analysis indicated a decrease in the phosphorylation level of both proteins in synemin null mice, and each play a role in cardiac contractility and hypertrophy (Dhalla and Müller, 2010; Rose et al., 2010; Mutlak and Kehat, 2015; Callaghan, 2016).

In light of the fact that increased phosphorylation of RII enhances affinity of the PKA holoenzyme for its AKAP (Zakhary et al., 2000), it is interesting that, in mice lacking synemin, there was a differential response with respect to the phosphorylation of RII. Specifically, there was an increase in the phosphoprotein in *skeletal* muscle and a decrease in *cardiac* muscle (Li et al., 2014; Garcia-Pelagio et al., 2018). These differences could be tissue-specific or due to differences in methods used to interrupt the synemin gene. The increase in RII phosphorylation in skeletal muscle was probably due to the increase in PKA activity in synemin null mice (Li et al., 2014). (PKA activity was not assayed in cardiac myocytes.) In each case, changes in RII phosphorylation may lead to changes in interaction between PKA and the other AKAPs. For example, since synemin is only one of approximately 17 AKAPs in cardiac myocytes (Kritzer et al., 2011), a decrease in RII α phosphorylation could result in reduced AKAP/PKA interaction and could be the cause of the reduction of PKA substrate phosphorylation. Furthermore, it is of particular note that AKAP-PKA interactions are altered in failing human hearts (Aye et al., 2012).

Researchers found that that synemin null mice had pronounced bone loss (Moorer et al., 2016). This was due to a reduction of osteoblasts leading to reduced bone formation. At a molecular level, synemin null mice exhibited a reduction of cyclin D1 mRNA expression in primary osteoblasts which coincided with enhanced osteogenic capacity. Interestingly, in primary osteoblasts from control mice, synemin was not in the IF network, instead it displayed punctate cytoplasmic staining. The authors speculated that the AKAP functionality of synemin might be responsible for the osteopenia because PKA plays a role in bone formation and osteoblast and osteoclast differentiation. However, it should be mentioned that the PI3K-Akt is also important in bone formation (Kawamura et al., 2007; Mukherjee and Rotwein, 2009; McGonnell et al., 2012).

It is important to note that a study assessing the role of synemin and vimentin in endothelial cells and vascular smooth muscle cells generated synemin null, vimentin null, and double knock-out mice (Langlois et al., 2017). The results indicate that the role of synemin in the parameters under study were practically all related to the functions of vimentin. Synemin null mice were not different from control animals. These findings strengthen the idea that the functions of synemin are tissue-specific.

### Binding Partners at the Sarcomere

In muscle cells, in addition to forming IF networks which are around the Z-disks, synemin binds to α-actinin, an integral component of the Z-disks. In these cells, α-actinin participates in cross-linking sarcomeric actin and linking the actin filaments to the costameres through its various other protein interactions (Capetanaki et al., 2007; Sjoblom et al., 2008; Henderson et al., 2017). Several studies have demonstrated that synemin binds α-actinin. Blot overlay and yeast two-hybrid studies showed that the binding site on chicken synemin is in the final 300 amino acids (aa 1292–1604) (Bellin et al., 1999, 2001). In neonatal cardiac myocytes, β-synemin co-immunoprecipitated with α-actinin and localized to Z-disks (Lund et al., 2012).

In human glioblastoma cell lines synemin co-localized with α-actinin at ruffled membranes, which are cellular domains active in cell motility. Interaction of the two proteins was further demonstrated by co-immunoprecipitation (Jing et al., 2005; Pan et al., 2008).

It should be noted that two studies probed for interaction between synemin and α-actinin via co-immunoprecipitation but were unable to identify any interaction (Uyama et al., 2006; Hijikata et al., 2008). The discrepancies in these findings with those described above could be due to differences in cell type and/or antibodies used.

Synemin has a second binding partner within the sarcomere. Specifically, α-synemin binds to the final domain of titin (the M10 region) positioning this isoform at the M-band of the sarcomere in cardiac muscle cells. The titin binding region of α-synemin is the final 102 amino acids of 312 amino acid insert that differentiates α- and β-synemin (Prudner et al., 2014). Titin, the largest protein in mammals, is a component of striated muscle, and spans half a sarcomere. Its N-terminal end is at the Z-disk and its C-terminal end is at the M-band. Functionally, it plays a role in the passive stiffness of cardiac myocytes (Hidalgo and Granzier, 2013). The role of α-synemin at the M-band is not known, but it is *not* likely to be related to a structural function based on the observation that down regulation of synemin did not result in disruption of M-band organization in neonatal cardiac myocytes (Lund et al., 2012). Additionally, in synemin null mice, the M-band in skeletal and cardiac myocytes developed normally (Li et al., 2014; Garcia-Pelagio et al., 2018). Even though titin itself is a PKA substrate, the phosphorylation sites are near the Z-disk end of the molecule, thus not likely to be substrates of α-synemin anchored PKA at the M-band.

### Binding Partners at Costameres/Focal Adhesions

Costameres (found in muscle cells) are akin to focal adhesion complexes in non-muscle cells. Costameres are located just below the sarcolemma in register with the Z-disk and, possibly, the M-band. IFs extend from the sarcomere to the costameres, linking the Z-disk to the sarcolemma. Within costameres are two protein complexes, the vinculin-talin-integrin system and the dystrophin-glycoprotein complex (DGC). Each provides a link from the interior of the cell to the extracellular matrix allowing for both “in-side out” and “out-side in” transfer of information (Ervasti, 2003; Peter et al., 2011; Cutroneo et al., 2012; Henderson et al., 2017). Interestingly, synemin is capable of binding proteins in each complex: vinculin and talin in the vinculin-talin-integrin complex (Bellin et al., 2001; Uyama et al., 2006; Sun et al., 2008b); and, α-dystrophin and α-dystrobrevin in the DGC (Mizuno et al., 2001; Bhosle et al., 2006).

Vinculin is expressed in all cells where it localizes to focal adhesions. Therefore, in muscle cells it is found at the costamere, specifically in the vinculin-talin-integrin complex. It links actin filaments to the sarcolemma. The vinculin binding site on synemin was localized to amino acids 1292-1604 in chicken synemin (Bellin et al., 2001). There are conflicting studies on the interaction between vinculin and mammalian synemin isoforms. In one study, human α-synemin was shown to interact with vinculin via blot overlays while β-synemin did not. In this study, the only region of interaction was mapped to a region found only in α-synemin, amino acids 1151–1462 (Figure 1). They also revealed interaction between α-synemin and metavinculin (a splice variant of vinculin) albeit, at a lower affinity than that for vinculin (Sun et al., 2008a). Immunostaining and co-immunoprecipitation in hepatic stellate cells also indicated interaction and co-localization of synemin and vinculin, the specific isoform of synemin was not confirmed in these studies (Uyama et al., 2006). However, in contrast to the first mammalian study described, β-synemin and vinculin were co-immunoprecipitated in two studies; specifically from rat skeletal muscle and rat neonatal cardiac myocytes (Hijikata et al., 2008; Lund et al., 2012). Additionally, Lund et al. (2012) did not detect interaction between synemin and metavinculin. These discrepancies could be due differences in methodologies, cell types, and/or cell development.

Talin mediates interaction between the extracellular matrix and the vinculin-talin-integrin complex. More specifically, it binds integrins and vinculin and thus links integrins to the sarcomere (Henderson et al., 2017; Klapholz and Brown, 2017). Studies in skeletal muscle and hepatic stellate cells showed synemin co-immunoprecipitated and co-localized with talin at costameres/focal adhesion complexes (Uyama et al., 2006; Sun et al., 2008b). The synemin binding site was localized to the α-synemin specific region, amino acids 1153–1464 (Figure 1; Sun et al., 2008b). Since this binding region had already been shown to be a binding site for vinculin, binding assays were carried out and it was determined that interaction within this region of synemin with vinculin and talin are mutually exclusive (Sun et al., 2008a, b).

Dystrophin is a large protein found in the DGC. Through its interaction with β-dystroglycan at the sarcolemma and actin filaments, dystrophin links the DGC to the cytoskeleton (Gao and McNally, 2015). Utrophin is a homolog of dystrophin found mostly in the DGC in neuromuscular junctions and myotendinous junctions in adult muscle (Perkins and Davies, 2002). The rod domain common to both synemin isoforms bound both utrophin and dystrophin in blot overlay and pull-down assays. Additionally, both utrophin and dystrophin bound to an α-synemin fragment containing the final 391 amino acids of C-terminal tail (Figure 1). Synemin and dystrophin co-localized in a rat smooth muscle cell line (Bhosle et al., 2006). The antibody used to detect synemin recognizes both isoforms. Thus, *in vivo*, it is unclear if dystrophin binds to both isoforms via the rod domain or just α-synemin through two interaction sites. It is tempting to speculate, based on the differential distribution seen in neonatal cardiac myocytes, in which α-synemin (but not β-synemin) was found at the sarcolemma and sites of cell-cell contact (Lund et al., 2012), that α-synemin is more likely to be found at the costameres due to multiple interaction sites.

In muscle cells, α-dystrobrevin is found in the DGC. It is part of a series of protein interactions that helps to hold the DGC together, the sarcoglycan-dystrobrevin-dystrophin complex (Gao and McNally, 2015). Yeast two-hybrid analysis revealed that β-synemin is able to bind α-dystrobrevin. Further analysis localized the synemin binding region to the rod domain (Mizuno et al., 2001). The two proteins co-localize in rat skeletal muscle (Hijikata et al., 2008). The ability of α-synemin to bind to α-dystrobrevin has not been tested, but, since the rod domain is identical in these two isoforms, it would be possible. Additionally, as previously mentioned, α-synemin has been shown to be the predominant isoform at the sarcolemma (Lund et al., 2012).

Zyxin is an LIM domain protein that shuttles from focal adhesions to the Z-disk and the nucleus. One of the many proteins it binds is α-actinin and its nuclear export signal facilitates its movements into and out of the nucleus. It is thought that zyxin responds to mechanical stress and then moves into the nucleus to activate gene expression related to cell survival (Brancaccio et al., 2006; Henderson et al., 2017). α- and β-synemin bind to the LIM domain repeats of zyxin. The binding region on synemin was mapped to the final ends of the C-terminal tails (α-synemin aa 920–1565, β-synemin aa 920–1253; Figure 1). Overexpression of the β-synemin binding region in three different cell types (HeLa, A-10, NIH 3T3) led to loss of zyxin at focal adhesions. When tested in HeLa cells, overexpression of a synemin peptide encompassing the zyxin binding region resulted in decreased adhesion and increased migration. While reduction of synemin via siRNA did not result in changes in zyxin localization, these cells did display significantly compromised adherence and migration. Therefore, it appears synemin does promotes adhesion and migration in HeLa cells; but, the role, if any, of zyxin in this function is still not clear (Sun et al., 2010).

### Other Binding Partners

Plectin 1 is an IF associated protein that, in general, connects all non-muscle cytoskeletal networks. In muscle cells it is found at various membrane locations such as desmosomes, intercalated discs, costameres and Z-disks. It works to anchor and organize IFs and interact with microtubules and actin microfilaments and also scaffold signaling proteins (Capetanaki et al., 2007; Wiche et al., 2015). Blot overlay assays probing for plectin 1 binding proteins detected β-synemin. The plectin binding site on β-synemin was localized to the region around the boundary of the rod and tail domain (Figure 1). α-synemin was not tested, but, as seen in Figure 1, the binding site is common to both isoforms so it is possible both can bind to plectin. Pull down assays and co-localization studies confirm interaction between β-synemin and plectin 1 in muscle cells. Immune complexes containing plectin, β-synemin, and components of the DGC and cytoskeleton (dystrophin, vinculin/metavinculin, α-dystrobrevin, desmin, and actin) were isolated from skeletal muscle (Hijikata et al., 2008).

Due to the unique web of interactions described above, synemin is well poised to crosslink the IF networks to the costameres, i.e., sites that are important for force transmission across the membrane, maintaining mechanical integrity of the membrane, and mechanotransduction (Sanghvi-Shah and Weber, 2017). Synemin is also well positioned to act as a crosslinker of IF networks and the sarcomeres via interaction with α-actinin (at the Z-disk) and titin (at the M-band) (Bellin et al., 2001).

### Role of Synemin as a Crosslinker

The role of synemin as a crosslinker was investigated in skeletal muscle using transgenic mice. The loss of interaction between synemin and its binding partners at the sarcolemma were thought to be the cause of membrane “wrinkles” observed in soleus muscle fibers (Li et al., 2014). Similarly, this loss of interaction is thought to be the cause of weaker costameres and a less stable sarcolemma in tibialis anterior muscle fibers (Garcia-Pelagio et al., 2015). In tibialis anterior muscles, these changes led to an increase in susceptibility of skeletal muscle to injury and, following injury, enhanced necrosis compared to controls. At the molecular level, the cause of the phenotype was proposed to be due to the loss of synemin binding to dystrophin and/or α-dystrobrevin; these proteins are found in the DGC and loss of DGC proteins leads to a similar outcome (Peter et al., 2011; Garcia-Pelagio et al., 2015).

## Synemin Tissue Distribution: Normal Tissue

Compared to other IF proteins, synemin has an unusually wide expression pattern. As most research is carried out in muscle and astrocytes, this section will discuss these tissues in depth and the others will be briefly mentioned.

### Muscle

Synemin was first identified in chicken smooth and skeletal muscle (Granger and Lazarides, 1980). It has since been found in all three muscle cell types in a variety of species (Bilak et al., 1998; Mizuno et al., 2001; Titeux et al., 2001; Hirako et al., 2003; Xue et al., 2004). Additionally, synemin is also found in myoepithelial tissue, a type of contractile muscle tissue which expresses cytokeratins rather than desmin, where it co-localizes with cytokeratin 5 and 6 as described above (Hirako et al., 2003; Noetzel et al., 2010).

With respect to isoform distribution, as expected, only one synemin isoform is expressed in chicken muscle. However, in mammalian muscle, isoform expression is tissue-specific. In adult tissue, Northern and Western analysis shows β-synemin is the predominant isoform in cardiac and skeletal muscle (Titeux et al., 2001; Hirako et al., 2003; Xue et al., 2004). Isoform expression in smooth muscle is more complex and varies with the type of smooth muscle. For instance, α-synemin was detected only in bladder while β-synemin was detected in bladder, blood vessels, and stomach (Xue et al., 2004). It is unclear which isoform(s) are expressed in myoepithelial tissue because synemin was detected via immunofluorescence using an antibody that recognizes both isoforms.

Interestingly, the SYNM gene is regulated by myocardin-related transcription factors in a serum response factor-independent manner (Sward et al., 2019). While this study focused on the role of myocardin-related transcription factors and synemin (among other proteins) in smooth muscle, it is worth mentioning that myocardin-related transcription factors also regulate skeletal and cardiac muscle development (Wang et al., 2001; Parmacek, 2007; Cenik et al., 2016). Whether or not these transcription factors regulate SYNM in striated tissue is currently unknown.

### Astrocytes

In addition to striated muscle, synemin has been extensively studied in astrocytes. Its expression is tissue and/or species specific as it is expressed only in astrocyte progenitor cells under normal conditions in rat and human brain (Sultana et al., 2000; Izmiryan et al., 2010). But it is expressed in mature astrocytes of the optic nerves in adult bovine and rabbit (Hirako et al., 2003). This difference may be species specific and/or attributed to the fact that the astrocytes in the optic nerve expresses vimentin in addition to GFAP. Synemin has been shown to have a preference for forming IFs with vimentin rather than GFAP in astrocytes as detailed above (Jing et al., 2007). Under normal conditions, synemin is not expressed in adult astrocytes, however, it is re-expressed in mature astrocytes in the human brain due to trauma or cancer (Jing et al., 2005). This is discussed in detail below in the section Changes in Synemin Expression: Astrocytes.

It is highly likely that α-synemin is the isoform expressed in astrocyte progenitor cells based on the molecular weight of the single band detected in those cells and the fact that the larger isoform is more abundant than the smaller one under pathophysiological conditions (Sultana et al., 2000; Jing et al., 2005).

### Other Tissues

Early research detected synemin in chicken erythrocytes and lens cells (Granger and Lazarides, 1980; Granger et al., 1982). In mammals, synemin has been discovered in lens cells, the retina, the CNS and PNS, hepatic stellate cells of the liver, pancreatic stellate cells (this was species specific, detected in rat and mice but not guinea pig), kidney mesangial cells, and peribronchiolar stellate-shapedfibroblasts. (Hirako et al., 2003; Tawk et al., 2003; Jing et al., 2005; Izmiryan et al., 2006, 2009, 2010; Schmitt-Graeff et al., 2006; Uyama et al., 2006; Mizuno et al., 2007, 2009; Luna et al., 2010). Synemin is also expressed in embryonic stem cells and adult muscle stem cells, i.e., satellite cells (de Souza Martins et al., 2011; Li et al., 2014; Mukund and Subramaniam, 2020).

## Synemin in Disease

Synemin expression is altered is several pathologies. The most extensive research in this field has been carried out with astrocytes. Recently, synemin mutations have been found in association with disease, in particular, heart disease.

### Changes in Synemin Expression: Astrocytes

Astrocytes exposed to trauma or disease become reactive astrocytes. This term encompasses a suite of changes including changes in gene expression and morphology (Hol and Pekny, 2015). The upregulation of the IF protein GFAP is the commonly used marker for reactive astrocytes, and now it is known that synemin is also upregulated in these cells (Jing et al., 2005). α-Synemin was upregulated and β-synemin, not seen in astrocyte progenitor cells, was also expressed in reactive astrocytes and astrocytoma cells (Jing et al., 2005; Luna et al., 2010). As discussed above, synemin was shown to have a role in glioblastoma cell proliferation due to its ability to bind PP2A and sequester it away from Akt (Pitre et al., 2012) and may have a similar role in these pathologies.

Alexander disease is a neurodegenerative disorder caused by mutations in GFAP that cause Rosenthal fibers to form in astrocytes. Synemin has been found in the Rosenthal fibers and in surrounding reactive astrocytes (Pekny et al., 2014). It is currently unknown if the protein aggregates formed by GFAP and synemin contribute to the pathology of the disease.

### Changes in Synemin Expression: Other Tissues

As mentioned earlier, synemin is found in hepatic stellate cells in the liver. Under pathological conditions it is upregulated in hepatic stellate cells and expression becomes more widespread in other cell types of the liver (Schmitt-Graeff et al., 2006). For example, during disease progression periportal fibroblasts become more synemin positive in more advanced fibrosis or cirrhosis. Synemin was also expressed in proliferating biliary epithelial cells and intrahepatic cholangiocarcinoma cells. This is unusual because synemin is not normally expressed in epithelial cells other than the contractile myoepithelial cells. The authors suggested that during liver disease these cells become more contractile-like.

The synemin gene was found to be one of the most frequently down-regulated genes in breast carcinoma tissue (Noetzel et al., 2010). Synemin is expressed in multiple locations including the myoepithelial cells of breast lobules and ducts in normal tissue. Very little or no synemin staining was detected via immunostaining in cancerous tissues. Methylation of the promoter was found to be the cause of synemin down-regulation. Loss of synemin correlated with unfavorable recurrence-free survival and positively with lymph node metastases and advanced tumor grade. Thus, it was proposed that loss of synemin production via methylation may be used as a predictive marker for risk of disease relapse.

A genome-wide expression profiling study using samples from patients with bladder exstrophy-epispadias complex identified 162 genes that were differentially expressed. Synemin was one of the two most significantly down-regulated genes (along with desmin) (Qi et al., 2011). Bladder exstrophy-epispadias complex is a spectrum of abnormalities where the distal urinary tract does not close during development. A significant portion of the dysregulated genes were associated with the cytoskeleton and desmosome leading the authors to suggest that the inability of IFs to anchor to the desmosome or abnormal desmosome formation may contribute to the disease.

### Synemin Mutations in Disease

To date, four mutations in the synemin gene (*SYNM*, GenBank reference sequences NM_145728 and NM_015286) have been linked to dilated cardiomyopathy (Zeller et al., 2006; Ware et al., 2016; Zhang et al., 2018). As shown in Figure 1, each of these mutations are located in the C-terminal tail of synemin. Specifically, two point mutations were found (c.1612T > C and c.2356G > A) which resulted in the missense mutations W538R and V746M (Zeller et al., 2006; Zhang et al., 2018). A deletion mutation (c.2576AGdel) was found that resulted in a frameshift mutation (818Tfs). Also, in the last exon, a point mutation (c.4647/3739C > T) resulted in a nonsense mutation (Q1519stop, α-synemin; Q1207stop, β-synemin). The location of the frame shift mutation and nonsense mutation is predicted to result in mutant (α and β) synemin protein that will have altered/missing binding sites for sarcomeric and costameric binding partners. Thus, these mutations could have multiple effects on synemin functionality, particularly its role in crosslinking. The functional outcome of the point mutations is less obvious since these mutations are not within any known binding sites. However, *in silico* analysis has revealed that missense mutation W538R is predicted to disrupt local residue interaction (Paulin et al., 2020). Specifically, replacement of tryptophan (a neutral apolar hydrophobic residue) with arginine (a positive hydrophilic residue) is predicted to result in loss of interaction between residue 538 and residue leucine 541. This loss would disrupt a salt-bridge stabilizing the local α-helix. The authors speculate that the proximity of this mutation to the PKA binding site would interfere with the ability of synemin to function as an AKAP.

A rare case of ulnar-mammary-like syndrome was found to be caused by a mutation in synemin (Zlotina et al., 2018). Ulnar-mammary-like syndrome is one of a group of poorly understood “heart-hand” type syndromes that affect both the heart and limbs. Typically, they are caused by mutations in transcription factors such as *TBX5*, *TBX3*, or *TFAP2B*, or an IF protein encoding nuclear lamins. In this study, the patient presented with cardiac dysfunctions including heart septal fibrosis and non-sustained left ventricular tachycardia. Additionally, she displayed pathology of the 5th digit, mental disability, and hypoplasia of the mammary glands. Due to this phenotype, the most likely causative mutation was predicted to be in *TBX3*, however this gene was not found to be altered. After the use of whole-exome sequencing and focusing on genes expressed in all tissues affected by the syndrome, 14 genes were identified. A mutant synemin gene was among this group and identified as the logical candidate gene. The point mutation (c.173C > T) resulted in the missense mutation A58V in the rod domain (Figure 1). This position is conserved across mammals. Studies in transgenic mice described above support the possibility of mutant synemin causing this condition. Specifically, in these animals, a lack of synemin led to heart defects and bone abnormalities. Mutation at this location not only might prevent synemin from inclusion in the IF network, but also from interaction with the other, non-IF binding partners that bind to the rod domain (Figure1).

## Conclusion

What is known about the role of synemin is evolving. It is an intermediate filament protein. It is a crosslinker. It is also a critical regulator of signaling pathways. Which functionality is dominant is isoform-, developmental-, and tissue-specific. What is clear is that it plays a critical role in the heart as emphasized by the clinical outcomes due to mutations in synemin and the results in mice lacking synemin. Work toward therapeutic applications using synemin will necessitate a better understanding of its different functions in an isoform-, developmental-, and tissue-specific manner.

## Author Contributions

MR wrote and edited the manuscript.

## Conflict of Interest

The author declares that the research was conducted in the absence of any commercial or financial relationships that could be construed as a potential conflict of interest.

## Abbreviations

aa: amino acid
AKAP: a-kinase anchoring protein
CDK: cyclin-dependent kinase
CDKI: cyclin-dependent kinase inhibitor
CNS: central nervous system
CREB1: cAMP Responsive Element Binding Protein 1
DGC: dystrophin-glycoprotein complex
GFAP: glial fibrillary acidic protein
IF: intermediate filament
IFAP: intermediate filament associated protein
mTOR: mechanistic target of rapamycin
PDK1: phosphoinositide-dependent kinase 1
PI3K: phosphoinositide 3-kinase
PIP2: phosphatidylinositol 4,5-bisphophate
PIP3: phosphatidylinositol 3,4,5-triphosphate
PKA: protein kinase A
PNS: peripheral nervous system
PP2A: protein phosphatase type 2A
Rb: retinoblastoma
RII α: type II regulatory subunit α of PKA
shRNA: short hairpin RNA.
